# Acute idiopathic polyradiculoneuritis with secondary arterial hypertension in a 5-year-old male Siberian Husky

**DOI:** 10.1080/01652176.2020.1820099

**Published:** 2020-09-18

**Authors:** Mihai Musteata, Raluca Ștefănescu, Andrei Radu Baisan, Diana Mocanu, Sorin Aurelian Pașca, Luminița Diana Hrițcu, Mădălina Henea, Gheorghe Solcan

**Affiliations:** a Department of Internal Medicine, Faculty of Veterinary Medicine, University of Agricultural Sciences and Veterinary Medicine” Ion Ionescu de la Brad” Iași, Iași, Romania; b Pathology Department, Faculty of Veterinary Medicine, University of Agricultural Sciences and Veterinary Medicine” Ion Ionescu de la Brad” Iași, Iași, Romania

**Keywords:** Polyradiculoneuritis, dog, arterial hypertension, heart rate variability

## Abstract

Acute canine idiopathic polyradiculoneuritis (ACIP) is one of the most common generalised neuromuscular diseases affecting dogs. In this report, we describe a 5-year-old, 25-kg, male, intact, Siberian Husky dog with ACIP with secondary induced arterial hypertension {systolic blood pressure [mean (m) ± standard deviation (sd)], 214 ± 19 mmHg; mean blood pressure (m ± sd), 164 ± 6.36 mmHg; and diastolic blood pressure (m ± sd), 137 ± 0.7 mmHg} and sinus tachycardia. Heart rate variability analysis indicated decreased vagal activity (low root-mean-square values of successive RR interval differences and percentages of the RR intervals differing by more than 50 ms in the entire recording) and predominance of sympathetic activity. Arterial hypertension was treated with amlodipine but remained greater than the upper limit for 51 days until the dog recovered ambulation. This is the first case report of ACIP and secondary arterial hypertension in a dog. Routine blood pressure measurements should be included in the monitoring of patients with ACIP if arterial hypertension might interfere with patient prognosis.

A 5-year old, 25-kg, male, intact, Siberian Husky dog was presented to the neurology service in the Veterinary Teaching Hospital Iași with acute non-ambulatory tetraparesis. The clinical signs occurred five days prior to presentation and consisted of progressive symmetrical weakness with onset from the posterior limbs that advanced to the anterior limbs. At presentation, neurological examination revealed areflexia and atonia in all four limbs with preserved perineal reflex, urinary continence, voluntary movements of the tail, and intact nociception. No signs of impaired cranial nerves were observed, but vocal changes over the last three days, suggesting dysphonia, were described by his owner. The dog could hold up his head and was able to eat and drink voluntarily. The rectal temperature was 38.6 °C, heart rate (HR) was 80 beats/min, and respiratory rate was 32 breaths/min. No recent illness, trauma, exposure to toxins, exposure to raccoon bites, or previous episodes of weakness prior to presentation were reported by the owner. Blood work revealed left-shift mild leukocytosis (15.3 G/L, reference value 5.0–14.1 G/L), an increased activity of creatine kinase (CK; 3454 U/L, reference value 57.9–239 U/L), and a modified albumin/globulin ratio (1.46, reference value 0.9–1.1). No blood gas analyses were performed.

The neuroanatomical localisation was consistent with a generalised lower motor neuron condition.

Cerebrospinal fluid tap, electrodiagnostic examination, and muscle biopsy (the owner declined nerve biopsy) were scheduled for the next day. Overnight, the dog developed tachycardia (140 beats/min) with short episodes of tachypnoea (up to 60 breaths/min) and was unable to hold up his head. The next day, the dog underwent abdominal ultrasonography and a complete cardiologic examination, which consisted of 5-min 10-lead electrocardiography (ECG; PolySpectrum 8 V ECG machine, Neurosoft, Ivanovo, Russia), trans-thoracic echocardiography (LogiqV5 ultrasound machine equipped with a 4–8-MHz phased-array probe, General Electric Medical Systems, Wuxi, China), thoracic radiography, and arterial blood pressure measurement (Thrall 2013; De Madron 2015; Tilley and Smith 2016).

The blood pressure was assessed noninvasively in the coccygeal artery with the dog positioned in lateral recumbency, using an oscillometric veterinary device equipped with a size-suitable cuff (VET HDO, S + B medVet, D2 cuff, Babenhausen, Germany). The mean value of five artefact-free measurements evaluated through the dedicated arterial pulse graphic was used for analyses (Brown et al. 2007). Blood pressure revealed high values: systolic blood pressure [SAP; mean (m) ± standard deviation (SD)] of 214 ± 19 mmHg, mean blood pressure (m ± SD) of 164 ± 6.4 mmHg and diastolic blood pressure (m ± SD) of 137 ± 0.7 mmHg. The HR variability (HRV) was analysed from the 5-min ECG recordings on days 2 and 8 following hospital admission. The RR intervals were transformed using ASCII text files and uploaded onto dedicated software (Kubios HRV v.2.1, Kubios, Kuopio, Finland) for further HRV analyses of the time and frequency domains (Malik et al. 1996).

Following the cardiologic examination, ECG revealed sinus arrhythmia, with an HR of 140 bpm, with a normal mean electrical axis and absence of a wandering pacemaker. No rhythm disturbances were present on the 5-min ECG recording. The ECG trace morphology was normal, except the P-wave, which was mildly increased in duration (60 ms) and did not change between the two ECG recordings.

Echocardiography revealed a normal left ventricular dimension, with a mildly decreased left ventricular internal diameter in diastole [1.11; 95% confidence interval (CI) 1.27–1.85] and normal value of left ventricular internal diameter in systole (0.71; 95% CI 0.71–1.26) (Cornell et al. 2004). The left atrium to aorta ratio was 1.33 (reference value <1.59) (Rishniw and Erb 2000). The valvular aspects and their kinetics were normal. The shortening fraction and ejection fraction were within normal ranges, with values of 32% (reference value 25–49%) and 62% (reference value 46.7–80.7%), respectively (Brown et al. 2003; Visser et al. 2019). An assessment of cardiac haemodynamics using Doppler revealed unremarkable results. Thoracic radiography revealed a normal pulmonary pattern, and the cardiac size was within the normal range (vertebral heart size 9.7 v, reference value 9.8 ± 0.6 v) (Greco et al. 2008). The first HRV analysis (on day 2 of hospitalisation) revealed autonomic nervous system (ANS) imbalance in the early stage of the disease reflected by a decrease in the overall time-domain HRV parameters (Baisan et al. 2020), suggesting a reduction in the vagal tone activity. These parameters are the most specific time-domain HRV measurements used to estimate vagally mediated changes (Shaffer and Ginsberg 2017). The abdominal ultrasound examination results were unremarkable.

The cerebrospinal fluid (CSF) tap, muscle biopsy, and electrophysiological tests were carried out under sedation with 0.03 mg/kg BW of medetomidine (Domitor, Pfizer, Espoo, Finland) and 0.1 mg/kg BW IV of ketamine (Ketamine, Kepro, Deventer, the Netherlands). The electrophysiological tests were performed using the Neuropack S, MEB 9400 K electrodiagnostic System (Nihon Kohden, Tokyo, Japan). The CSF was considered normal in terms of the number of cells (3.3 cells/µL, reference value <5 cells/µL (Di Terlizzi and Platt 2009)) and protein level (148.7 mg/L, reference value <300 mg/L (Di Terlizzi and Platt 2009)). On electromyography examination, abundant spontaneous activity (fibrillation potentials and positive sharp waves) were observed both in the pelvis and thoracic limb musculature (+3 on the Kimura scale (Kimura, XXXX)). However, analysis of individual motor unit action potentials was not performed. Motor nerve conduction velocity (MNCV) measurements were performed for the peroneal, tibial, and radial nerves based on recordings from the plantar, palmar interosseous, and extensor carpi ulnaris muscles, respectively. Severe reductions in the MNCV in all examined nerves (peroneal 37.8 m/s, tibial 19.1 m/s, radial 31.5 m/s; reference value >60 m/s) (Cuddon 2002) and an increase in the measured F-wave latency (26.2 m/s for the tibial nerve) compared to the expected F-wave latency (lower than 13 m/s (Cuddon 2002)) were observed. The findings of a sensory nerve conduction study and repetitive stimulation were normal. Two muscle samples were obtained from the quadriceps and cranial tibial muscle and were submitted for histological examination. Both samples were embedded in paraffin and stained using the Masson trichrome method, and no pathological features were observed when analysed by an experienced pathologist (S.A.P).

A clinical diagnosis of acute polyradiculoneuritis, arterial hypertension, and sinus tachycardia was made.

For the next 14 days, the dog was hospitalised for physiotherapy; blood pressure monitoring; and daily HR, respiratory rate, and HRV studies. The HR, respiratory rate and SAP for the first 15 days of hospitalisation are presented in Figure 1. To reduce the arterial blood pressure and avoid target organ damage, the calcium channel blocker amlodipine was administered (0.2 mg/kg BW/day *per os*; Amodip, Ceva Santé Animale©, Louverné, France) (Acierno et al. 2018). The blood pressure decreased immediately but remained greater than the upper limit. In contrast, the HR progressively increased during the monitoring period (Figure 1). On day 8 of hospitalisation, the HRV based on overall time-domain analysis revealed an even more severe sympathovagal imbalance expressed by reduced SDs of the averages of RR intervals compared to those on day 2. Moreover, vagal tone indicators such as the root-mean-square of successive RR interval differences, percentages of the RR intervals differing by more than 50 ms in the entire recording, and high-frequency (HF) band were also decreased on day 8 compared to those on day 2. Between the two HRV studies, the low frequency (LF)/HF ratio increased by 57% due to the marked decrease in the vagal activity. Percentage variations in HRV time- and frequency-domain parameters are presented in Table 1.

**Figure 1.**
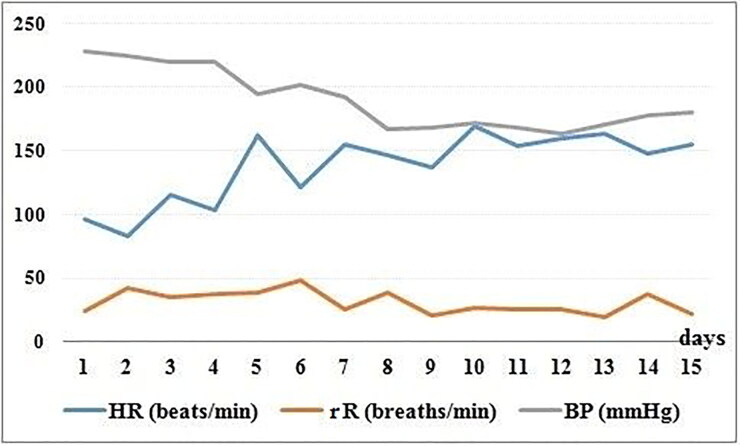
Heart rate, respiratory rate, and systolic blood pressure course during the hospitalisation period (2 weeks). The values were recorded in the morning (between 8 and 9 a.m.). HR, heart rate (beats/min); rR, respiratory rate (breaths/min); BP, blood pressure (mmHg). Horizontal axis, days.

**Table 1.** Heart rate variability parameters derived from five-minute electrocardiographic traces recorded on days 2 and 8 of hospitalisation.

		Day 2	Day 8 (percentage change compared to day 2)	Reference values 95% CI
Time-domain HRV results	Mean RR (ms)	430	500 (+16.2%)	471–519
	SDNN (ms)	55.5	37.6 (-32,2%)	60–77.1
	rMSSD (ms)	42.9	30.5 (-28,9%)	69.9–102.5
	pNN50% (%)	24.1	9.8 (-59,3%)	29.2–41.7
Frequency-domain HRV Results	LF (ms^2^)	575	162 (-71,8%)	714–1113
	HF (ms^2^)	713	128 (- 82,4%)	932–2385
	LF/HF	0.80	1.26 (+ 57,5%)	1.07–1.8

CI, confidence interval; HRV, heart rate variability; mean RR (ms), mean value of the time intervals between consecutive heart beats; SDNN, standard deviation of the averages of RR intervals, rMSSD, root-mean-square of successive RR interval differences; pNN50%, percentages of the RR intervals differing by more than 50 ms in the entire recording; LF, low frequency power expressed as ms^2^; HF, high frequency power expressed as ms^2^; LF/HF, low frequency/high frequency ratio; reference values according to literature [11]

From the second week of hospitalisation, the patient could lift his head for a short time. After 15 days of hospitalisation, due to the stable condition of the patient and his owner’s financial aspects, the owner decided to continue the physiotherapy procedures and medication at home. After discharge, the blood pressure was monitored once per week (by one of the authors: DM) using the same technique and device used previously. The voluntary movements of the limbs appeared after 28 days, and the dog became ambulatory without support 51 days after initial presentation. The SAP was in the normal range from that day, and the amlodipine dosage was reduced for another 14 days to 0.1 mg/kg BW/day and then discontinued. Complete recovery in the dog was noticed 9 weeks after initial presentation. Further follow-ups were scheduled (3, 4, 6, 9, and 14 months after ambulatory status), and no signs of arterial hypertension were noted. The changes in blood pressure from presentation to day 80 are presented in Figure 2.

**Figure 2.**
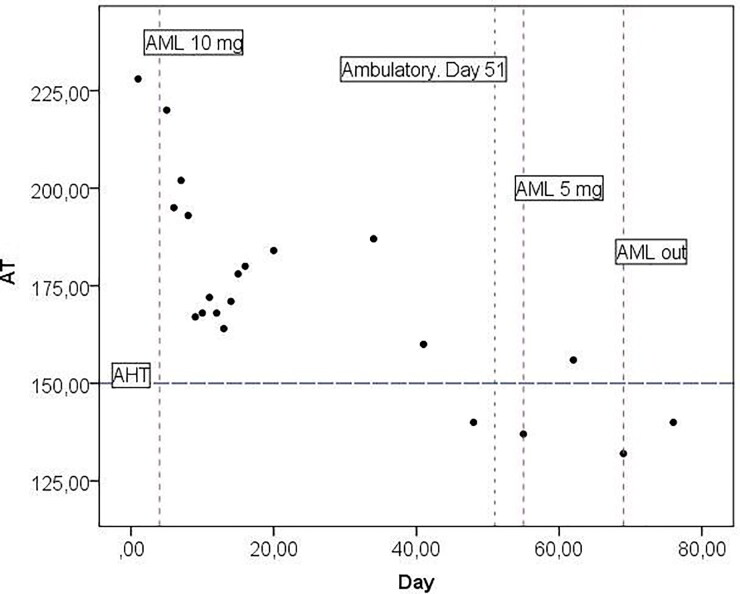
Change in systolic blood pressure over time until day 80 from commencement of treatment. AML, amlodipine; AML out, withdrawal of amlodipine; AT, systolic arterial blood pressure (mmHg); AHT (horizontal dashed line), threshold for systemic arterial hypertension. Vertical dashed lines, onset and changes in AML dosage and onset of the ambulatory status (day 51).

Acute idiopathic polyradiculoneuritis (ACIP) is the most common generalised polyneuropathy to occur in dogs. The clinical presentation is usually characterised by acute onset of progressive symmetrical ascending weakness expressed by hypo- or areflexia associated with normal sensory function (Laws et al. 2017; Martinez-Anton et al. 2018). In most cases, clinical signs are limited to the limbs, but involvement of the cranial nerves (including recurrent laryngeal nerve involvement causing dysphonia) or respiratory paresis or paralysis caused by intercostal or phrenic nerve involvement has been reported (Anor 2014). Patients with cranial nerve impairment can further develop *ab ingestis* pneumonia. Both patients reported with intercostal muscle malfunction and *ab ingestis* pneumonia needed specific intensive care procedures [19]. In the present case, during the first night of hospitalisation, the patient developed episodes of tachypnoea, but no signs of bronchopneumonia were noticed on radiological examination, and no intercostal muscle impairment (such as visible chest movements during breathing) was observed.

In patients with ACIP, blood test results are usually either unremarkable or nonspecific unless respiratory paralysis occurs, which may be reflected in abnormal blood gas levels (Anor 2014). In this case, blood gas analyses were not performed, but no signs of laboured breathing were noted. In a study of 27 dogs with ACIP, the CK activity was increased in 22% and thyroxine level was decreased in 18% of the patients. In the same study, the authors found albuminocytologic dissociation in the CSF in 43% of the analysed samples (Martinez-Anton et al. 2018). In our patient, we found only an increased activity of CK (without associated histopathological findings) and no changes on CSF analysis. In ACIP, the most common electrophysiological features are represented by spontaneous muscle activity (Martinez-Anton et al. 2018), but reduced MNCV is sometimes described (Drobatz et al. 2019), both of which were observed in our patient. On ECG, only the P-wave duration showed mildly increased values. This finding may suggest left atrial enlargement, since the P-wave duration reflects activation of the atrial muscle, which primarily depends on the mass of tissue excited (Soto-Bustos et al. 2017). However, a previous study showed that the diagnostic performance of the P-wave duration for identification of left atrial enlargement in dogs presents considerable limitations (Savarino et al. 2012). Furthermore, in the present case, the left atrium dimension was within the normal range based on echocardiographic measurement.

Based on the medical history, acute onset of weakness, and results of clinical findings and additional examinations, we suspected ACIP as the most likely diagnosis for our patient. Usually, the prognosis of patients with ACIP is good with a high proportion of patients recovering in weeks. Treatment is usually restricted to physical rehabilitation and supportive care, but a faster recovery rate was reported in sixteen dogs with ACIP after intravenous immunoglobulin administration (Hirschvogel et al. 2012). Different manifestations of ACIP and other neuromuscular conditions have been reported in human and animals (Zhou et al. 2013; Stanciu and Solcan 2016). To the best of our knowledge, this is the first case of arterial hypertension associated with ACIP in a dog. The dog had no history of arterial hypertension and/or cardiorespiratory impairments prior to the ACIP occurrence.

Differential diagnoses for arterial hypertension in dogs include heart failure, acute or chronic kidney failure, hyperadrenocorticism, diabetes mellitus, obesity, pheochromocytoma, primary hyperaldosteronism, hypothyroidism, and idiopathic arterial hypertension (Acierno et al. 2018). Based on clinical findings and additional examinations, all of the aforementioned aetiologies were excluded with the exception of hypothyroidism and primary hyperaldosteronism. However, an association between ACIP and a hypothyroid condition is uncommon in dogs (Acierno et al. 2018), and the patient did not present with any clinical signs of hypothyroidism. Primary hyperaldosteronism was excluded based on routine blood work, normal abdominal ultrasound and no ECG abnormalities consistent with hypokalemia on multiple examinations. Therefore, idiopathic arterial hypertension remained the most likely differential diagnosis by exclusion.

In humans, autonomic dysfunction related with acute inflammatory demyelinating polyneuropathy includes transient arterial hypertension or, less often, hypotension, sinus tachycardia, bradycardia, and urinary retention, which usually improves in parallel with motor and sensory function (Cuciureanu and Prodan 2004). In our patient, the blood pressure progressively decreased after the amlodipine administration, as expected. The increase in the HR may have been a consequence either of the early sympathetic activation or amlodipine administration (Kuramoto et al. 2003). Arterial hypertension induced by acute polyradiculoneuritis has been reported in two-thirds of paediatric patients with Guillain–Barré syndrome (GBS) and up to 75% of affected adults (Pfeiffer 1999; Dimario and Edwards 2012; Samadi et al. 2013; Walling and Dickson 2013; Tanaka and Satomi 2016). Furthermore, in paediatric patients with GBS, arterial hypertension may be a prominent early-stage feature, and urinary normetanefrine can increase to levels observed in pheochromocytoma, which may create diagnostic confusion (Abdel-Salam et al. 2013). In adult patients with GBS, plasma noradrenaline, dopamine, and urinary noradrenaline levels have been reported to be increased (Tanaka and Satomi 2016). Other autonomic dysfunctions such as bladder storage dysfunction, pulmonary hypertension, orthostatic hypotension, sinus tachycardia, and sinus arrest have also been reported in humans (Pfeiffer 1999; Rooney and Thomas 2010).

In our patient, HRV analysis was used to identify the ANS imbalance. We suggest that the patient developed arterial hypertension secondary to the ANS imbalance expressed as a vagal decrease with a sympathetic tone predominance as reflected by the HRV analyses. The tension of vascular smooth muscle cells is maintained by sympathetic nerve activity and opposed by endothelial relaxing factors such as nitric oxide (Berg and Jensen 2011). The HRV analyses showed evidence of an ANS imbalance represented by vagal reduction and sympathetic overactivity. A study in humans with GBS demonstrated similar results in terms of frequency-domain parameters. In 1997, Flachenecker et al. found that when the disease was the most severe, the HF component is significantly decreased, while the LF/HF ratio, which has been suggested to be an indicator for sympathetic activity, is increased compared with the follow-up values after one year (Flachenecker et al. 1997). Another study performed in humans with hypertension found that vagal withdrawal is more prominent than sympathetic overactivity. It was concluded that vagal inhibition plays an important role in the critical alterations in sympathovagal balance in the development of clinical arterial hypertension (Pal et al. 2011). Another underlying mechanism involves baroreceptor deafferentation, resulting in resting tachycardia and arterial hypertension, and this may explain the sympathetic hyperactivity in humans with GBS (Fagius et al. 1985). Based on the assumption that vagal activity is physiologically closely related to the sympathetic arm (Malik et al. 1996), it is tempting to speculate that uncontrolled sympathetic overactivity may induce increased vascular resistance, resulting in systemic arterial hypertension.

Interestingly, in the present case, within a few days prior to the dog becoming ambulatory, the blood pressure returned to normal and remained within the normal range. Based on the disease progression, we suspected that the arterial hypertension was induced by the ACIP likely sharing the same pathogenesis as that described in humans. This may be explained by the fact that blood pressure variability can be attributed to disturbances in the baroreceptor reflex pathway, as well as changes in catecholamine levels (Gupta et al. 2020).

In summary, we reported a case of ACIP with autonomic disturbances (arterial hypertension and sinus tachycardia) that disappeared once the neuropathy was resolved. The HRV analysis was proven to be reliable to assess ANS imbalance associated with ACIP in dogs. Routine blood pressure measurements should be included in the monitoring of patients with ACIP if the complications (eye haemorrhage, retinal detachment, stroke, etc.) might interfere with patient prognosis.
